# A case of pulmonary thromboembolism due to coagulation factor V Leiden in Japan ~ usefulness of next generation sequencing~

**DOI:** 10.1186/s12959-017-0132-6

**Published:** 2017-03-14

**Authors:** Daisuke Sueta, Miwa Ito, Mitsuhiro Uchiba, Kenji Sakamoto, Eiichiro Yamamoto, Yasuhiro Izumiya, Sunao Kojima, Koichi Kaikita, Satoru Shinriki, Seiji Hokimoto, Hirotaka Matsui, Kenichi Tsujita

**Affiliations:** 1 Department of Cardiovascular Medicine, Graduate School of Medical Sciences, Kumamoto University, 1-1-1, Honjo, Chuo-ku, Kumamoto, 860-8556 Japan; 2 Blood Transfusion and Cell Therapy, Kumamoto University Hospital, Kumamoto, Japan; 3 Department of Molecular Laboratory Medicine Graduate School of Medical Sciences, Kumamoto University, Kumamoto, Japan

**Keywords:** Thromboembolism, Leiden mutation, Next generation sequencer

## Abstract

**Background:**

Because the venous thromboembolisms (VTEs) due to the coagulation factor V R506Q (FV Leiden) mutation is often seen in Caucasians, the VTE onset in Japan has not been reported.

**Case presentation:**

A 34-year-old man from north Africa experiencing sudden dyspnea went to a hospital for advice.

The patient had pain in his right leg and a high plasma D-dimer level. A contrast-enhanced computed tomography scan revealed a contrast deficit in the bilateral pulmonary artery and in the right lower extremity. The patient was diagnosed with VTE, and anticoagulation therapy was initiated. Our targeted gene panel sequencing revealed that the occurrence of VTE was attributed to a presence of the FV Leiden mutation.

**Conclusions:**

This is the first report demonstrating VTE caused by the FV Leiden mutation in Japan.

## Background

Because the causes of venous thromboembolisms (VTEs) are various, it is important to differentiate inherited thrombophilia. The coagulation factor V R506Q (FV Leiden) mutation, which is known to be a cause of VTE, is often seen in Caucasians, whereas the mutation has not been reported in the Japanese population [1, 2]. We present the first case of VTE caused by coagulation FV Leiden mutation in Japan, demonstrating the usefulness of next generation sequencing (NGS).

## Case presentation

**Fig. 1:**
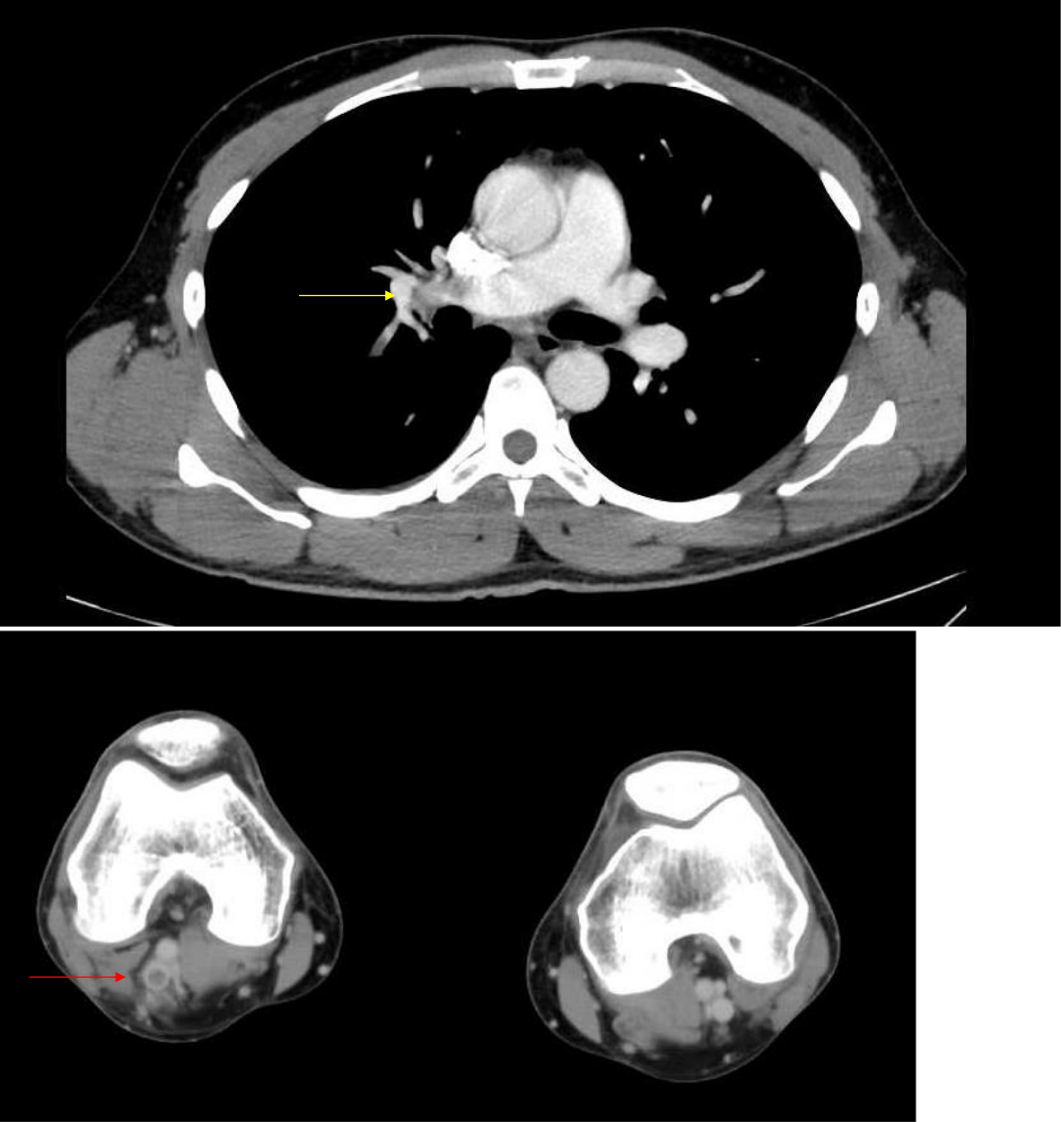
Contrast-enhanced computed tomography at the first visit. **a** A contrast deficit in the bilateral pulmonary artery (*yellow arrow* indicates the right pulmonary artery). **b** A contrast deficit in the left lower extremity (*red arrow*)

A 34-year-old previously healthy and nonsmoking man without any history of VTE or renal disease visited an emergency department (ED) because of a complaint of sudden dyspnea at rest and right lower extremity pain. He was Caucasian and from north Africa; his grandfather was from the Netherlands. There was no family histories of thrombosis. He did not have any VTE risks, such as obesity, cancer, surgery, immobilization or recent long travel. Two weeks before the visit, he fell off his bicycle during mountain climbing and strongly hit his right calf. His plasma D-dimer level in the ED was 31.6 μg/mL, and deep vein thrombosis (DVT) was suspected. A contrast-enhanced computed tomography (CT)-scan revealed a contrast deficit in the bilateral pulmonary artery (Fig. 1a) and in the right lower extremity (popliteal vein) (Fig. 1b). He was diagnosed with VTE and admitted to our department. On admission, his blood pressure was 133/72 mmHg, and his heart rate was 110 beats per minute. His degree of oxygen saturation (SaO2) on an arterial blood gas was 98%. His body mass index was 21.3 kg/m2. Although multiple VTEs in the bilateral pulmonary arteries were observed, electrocardiography and cardiac ultrasound did not indicate heart failure. His right ventricular function was intact (estimated pulmonary artery pressure = 32/18 mmHg on cardiac ultrasound). His plasma brain natriuretic peptide and high-sense troponin T concentration levels were less than 5.8 pg/mL and 0.004 ng/mL, respectively. Because of his recent history of a trauma, thrombolytic therapy was not recommended; therefore, anticoagulation therapy using heparin was initiated. Heparin was switched to a direct oral anticoagulant (DOAC); 20 mg daily administration of apixaban (trade name: EliquisTM, Pfizer) was initiated according to the results of the AMPLIFY trial [3].

**Fig. 2:**
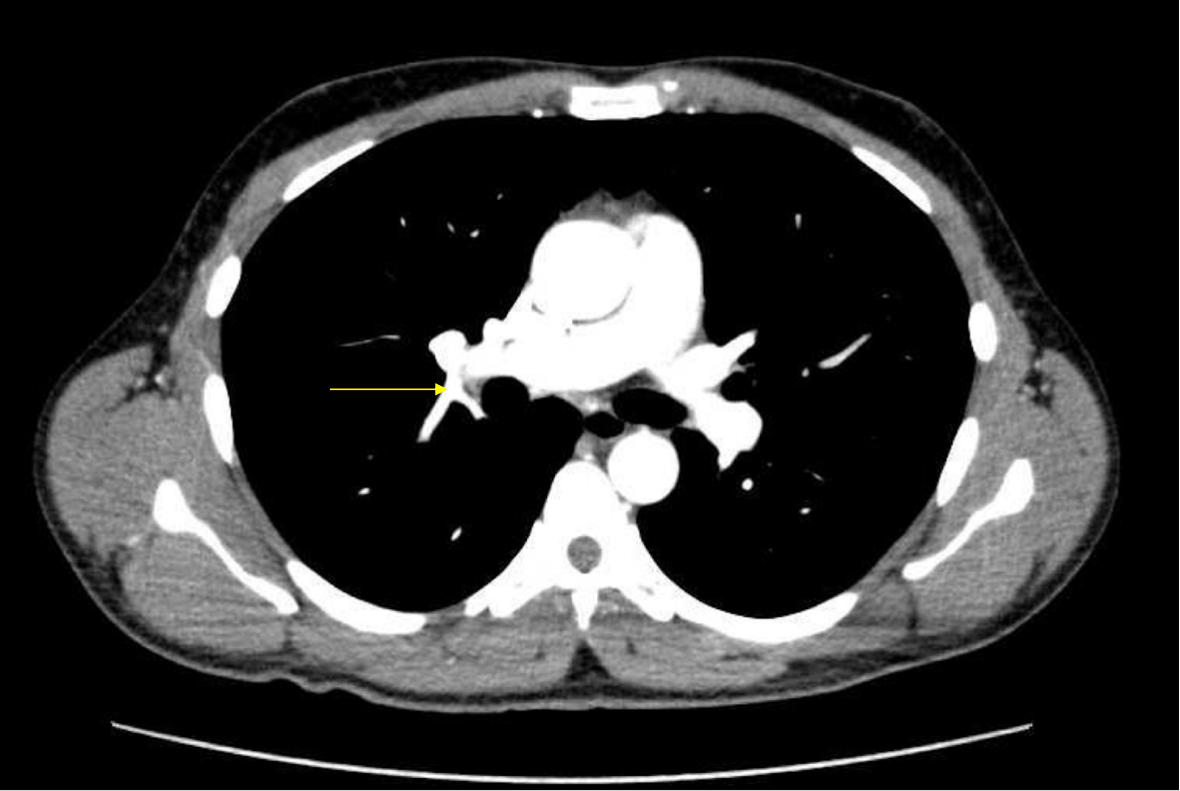
Contrast-enhanced computed tomography 7 days after the initiation of anticoagulation therapy. The *yellow arrow* indicates reduced thrombosis

An ordinary investigation of most factors related to thrombus formation (including protein C (82%), protein S(92%), antithrombin(80%), and antiphospholipid antibodies(<8U/mL)) revealed that the blood concentrations of all such factors were within the reference range. The protein C and antithrombin were measured using commercially available reagents (Testzym®S PC for protein C, Testzym®S ATIII for antithrombin, Sekisui Medical Co., Tokyo, Japan) according to the protocol supplied by the manufacturer. Protein S (latex agglutination reaction) and antiphospholipid antibodies (ELISA) measurements were performed at SRL Inc., Tokyo, Japan. Therefore, we attempted to screen for the genetic cause of the VTE using NGS technology. We had designed a screening panel of genes for use with the Illumina TruSeq Custom Amplicon platform (Illumina, Inc., San Diego, CA, USA). The panel includes amplicons defining all coding exons of the genes mutations that are known to cause VTE: F5, SERPINC1, PROC, PROS1 and F2. Sequencing was performed using the MiSeq Illumina sequencer (Illumina, Inc.). A total of 14,828 base pairs were sequenced per sample from 107 amplicons. The obtained sequences were aligned to the reference genome (GRCh37̸hg19) using MiSeq Reporter software (Illumina, Inc.). A vcf file containing variant calls was generated, further reviewed, and filtered. The variant frequency which is the ratio of the sum of the called variant (C > T) depth to the total depth was 0.478 as assessed by NGS. Thus, we identified the heterozygous FV Leiden mutation. Therefore, we concluded that the FV Leiden mutation was the primary cause of the VTE. Then, according to a previous report [4], we continued treatment with DOAC. After 7 days, a follow-up contrast-enhanced CT scan revealed a reduction in the thrombosis (Fig. 2). The patient was followed up with apixaban.

The patient gave his consent for the publication of this study.

## Discussion and conclusions

Recently, DVT has been focused on and recognized by medical professionals and by general citizens. In general citizens, long flight thrombosis, so-called “economy class syndrome”, and VTEs caused by sleeping in a vehicle after a disaster [5] have been reported by mass media. In medical professionals, mechanical compressions [6], malignant diseases [7, 8], lower limb operations [9], sedentary postures for long periods and central vein catheters cause VTEs, and DVT often results in fatal pulmonary thromboembolism. Generally, as globalization has advanced in Japan, flexibility in diagnosis and treatment is becoming increasingly necessary.

In 1993, Dahlbäck and colleagues reported familial thrombophilia due to a resistance to activated protein C [10]. This abnormality is caused by the substitution of a single amino acid—glutamine for arginine—at position 506 (arginine506 → glutamine) in the coagulation factor V molecule [11]. This mutated factor is also referred to as FV Leiden (R506Q), which is named after a city in the Netherlands that has many family lineages [12]. This mutation is found in approximately one fifth of patients with venous thromboembolism in western countries [11], and it has a much stronger association with DVT than with VTE; this observation is called the Leiden paradox [13]. The risk of recurrent thromboembolic events is significantly higher in carriers of FV Leiden than in patients without this abnormality [11]; however, FV Leiden is not reported to be a strong predictor of recurrent VTE [14].

In this case, the patient was young and healthy, had no past history and was not taking any regular medications. In addition, the trauma in right lower extremity was relatively mild. Therefore, the occurrence of VTE was thought to be triggered by a trauma in addition to genetic background. DOAC is effective for the treatment of VTE, in addition to the prevention of brain infarction in atrial fibrillation [15]. Some recent reports demonstrated that direct thrombin inhibitors (dabigatran, argatroban, and bivalirudin) [16] and a DOAC (rivaroxaban) [4] are effective for VTE caused by FV Leiden; this case revealed apixaban was also effective.

NGS has revolutionized genetic research and the molecular diagnosis of human genetic disease [17]. Targeted gene panel sequencing provides enhanced sequencing depth and can avoid incidental findings while being cost-effective compared with sequencing the exome [18]. In this case, our customized panel targeting congenital coagulation defects enabled the rapid detection of the causative gene mutation, which has not been identified in the Japanese population with high accuracy. Therefore, the technology may be useful for the clinical diagnosis of patients with suspected congenital coagulation defects without regard to race, which could facilitate effective diagnosis.

To the best of our knowledge, this case represents the first report of a VTE caused by FV Leiden in Japan. The present report suggests that all medical professionals in Japan should be aware of the presence of this mutation.

## Abbreviations

CT: Computed tomography
DOAC: Direct oral anticoagulant
DVT: Deep vein thrombosis
ED: Emergency department
FV: Coagulation factor V
NGS: Next generation sequencing
VTE: Venous thromboembolism
